# Catalase down-regulation in cancer cells exposed to arsenic trioxide is involved in their increased sensitivity to a pro-oxidant treatment

**DOI:** 10.1186/s12935-018-0524-0

**Published:** 2018-02-20

**Authors:** Christophe Glorieux, Pedro Buc Calderon

**Affiliations:** 1 Metabolism and Nutrition Research Group, Louvain Drug Research Institute, Université catholique de Louvain, MNUT 7309, LDRI, UCL Avenue E. Mounier 73, 1200 Brussels, Belgium; 2 Facultad de Ciencias de la Salud, Universidad Arturo Prat, 1100000 Iquique, Chile; 3 Present Address: State Key Laboratory of Oncology in South China, Collaborative Innovation Center of Cancer Medicine, Sun Yat-Sen University Cancer Center, Guangzhou, 510275 China

**Keywords:** Catalase, Arsenic trioxide, Pro-oxidant therapy, Breast cancer cells

## Abstract

**Background:**

Pro-oxidant drugs have been proposed for treating certain cancers but the resistance developed by cancer cells to oxidative stress limits its potential use in clinics. To understand the mechanisms underlying resistance to oxidative stress, we found that the chronic exposure to an H_2_O_2_-generating system (ascorbate/menadione, Asc/Men) or catalase overexpression (CAT3 cells) increased the resistance of cancer cells to oxidative stress, likely by increasing the antioxidant status of cancer cells.

**Methods:**

Modulation of catalase expression was performed by either protein overexpression or protein down-regulation using siRNA against catalase and aminotriazole as pharmacological inhibitor. The former approach was done by transfecting cells with a plasmid construct containing human catalase cDNA (CAT3 cells, derived from MCF-7 breast cancer cell line) or by generating resistant cells through chronic exposure to an oxidant injury (Resox cells). Cell survival was monitored by using the MTT reduction assay and further calculation of IC_50_ values. Protein expression was done by Western blots procedures. The formation of reactive oxygen species was performed by flow cytometry. The transcriptional activity of human *catalase* promoter was assessed by using transfected cells with a plasmid containing the − 1518/+ 16 promoter domain.

**Results:**

Using Resox and CAT3 cells (derived from MCF-7 breast cancer cell line) as models for cancer resistance to pro-oxidative treatment, we found that arsenic trioxide (ATO) remarkably sensitized Resox and CAT3 cells to Asc/Men treatment. Since catalase is a key antioxidant enzyme involved in detoxifying Asc/Men (as shown by siRNA-mediated catalase knockdown) that is overexpressed in resistant cells, we hypothesized that ATO might regulate the expression levels of catalase. Consistently, catalase protein level is decreased in Resox cells when incubated with ATO likely by a decreased transcriptional activity of the *catalase* promoter.

**Conclusions:**

Our findings support the proposal that ATO should be administered in combination with pro-oxidant drugs to enhance cancer cell death in solid tumors.

## Background

Arsenic has long been known as a poison and environmental carcinogen affecting high number of people [1]. Nevertheless, its remarkable effect in the treatment of acute promyelocytic leukemia (APL) has made its mechanism of action an intense issue of interest in cancer therapy. Particularly, the administration of arsenic trioxide (ATO) alone or in combination with either retinoic acid (ATRA) or chemotherapeutic drugs has emerged as an effective option to treat newly diagnosed and relapsed APL patients [2–5]. Its therapeutic effect has been also observed in various solid tumors including breast cancer [6–8].

The mechanism of action of ATO is complex; it can induce cell apoptosis, cellular differentiation, and inhibition of NF-κB activity in APL patients [9]. In addition, the mechanisms of ATO cytotoxicity also rely in degradation of the leukemia fusion protein, namely PML-retinoic acid receptor alpha (PML-RARα) restoring PML functions [10, 11]. Some lines of evidence suggest that ATO acts via the formation of reactive oxygen species (ROS) due to NADPH oxidase activation [12]. Moreover, ATO leads to decreased levels of glutathione (GSH) and inhibits the antioxidant activity of enzymes like glutathione peroxidases (Gpx), glutathione S-transferases and catalase [13]. ATO also has high affinity for sulfhydryl groups in redox-sensitive proteins likely explaining many of its ROS-related effects [9].

Given the growing interest to use pro-oxidant cancer therapies, the risk to develop cancer cell resistance against an oxidative stress may be a major obstacle. To study this issue, we have generated MCF-7 cells where catalase was overexpressed by plasmid transfection, namely MCF-7 CAT3 cells [14]. In such cells, we have observed an increased resistance against pro-oxidant drugs (acting mainly by H_2_O_2_-mediated processes), while the expression levels of Gpx and Peroxiredoxin 2 (Prdx2) were decreased by ~ 40% [14]. Therefore, the question about a potential upregulation of catalase during chronic, sub-lethal exposures to pro-oxidant treatment was addressed. Thus, we explored the role of signaling pathways and transcription factors in the regulation of *catalase* gene expression [15]. We found that Akt/PKB signaling is a repressive pathway that decreases catalase expression [16, 17]. In addition, we demonstrated the important roles of RARα and JunB transcription factors in remodeling the chromatin and controlling catalase expression in breast cancer cells [16, 17].

Pro-oxidant chronic treatments may lead cancer cells to acquire resistance against oxidative stress by overexpressing catalase and other antioxidant enzymes. Therefore, we generated Resox cells, a MCF-7 cell line resistant to pro-oxidant treatments [16–19] as cell model for studying the mechanisms underlying cell resistance to pro-oxidant treatment.

The aim of this work was to study a potential link between ATO and catalase expression and the functional consequences of such putative relationship in mammary cancer cells exposed to ascorbate/menadione (Asc/Men), a H_2_O_2_-generating system widely used to induce oxidative stress [20, 21]. To this end, we employed the following mammary cells: normal non-tumor epithelial breast cell line (250MK), the breast MCF-7 cancer cell line, and the MCF-7 cells in which catalase was overexpressed either by plasmid transfection (CAT3 cells) or by chronic exposure to a pro-oxidant treatment (Resox cells).

## Methods

### Cell culture and chemicals

Human breast cancer cell line MCF-7 was purchased from ATCC (Manassas, VA, United States). Cells were maintained in DMEM medium supplemented with 10% fetal calf serum, penicillin (100 U/ml) and streptomycin (100 µg/ml) from Gibco (Grand Island, NY, USA). Human mammary epithelial cells 250MK were provided by Dr. M. Stampfer and J. Garbe (Lawrence Berkeley National Laboratory, Berkeley, California, USA). They were maintained in a M87A + CT + X medium and used between passages 8–10 [22]. The cultures were maintained at a density of about 50 × 10^3^ cells/cm^2^. All cultures were maintained at 37 °C in 95% air/5% CO_2_ with 100% humidity.

### Cell treatments

Cancer cell cultures were treated with sodium ascorbate and menadione sodium bisulfite (Asc/Men) in a ratio of 100/1 (ascorbate in mM/menadione in µM), arsenic trioxide (5–10 μM), hydrogen peroxide (0–1 mM), or 5 mM of 3-amino-1,2,4-triazole (ATA). All reagents were purchased from Sigma-Aldrich (St Louis, MO).

### Generation of MCF-7 cell line stably expressing catalase

Breast cancer cell line MCF-7 CAT 3 overexpressing catalase was established from wild-type MCF-7 cell line, which was purchased from ECACC (Salisbury, United Kingdom). Plasmid construct pZeoSV2(1) containing human catalase cDNA was a kind gift from Professor A. Cederbaum (Mount Sinai Hospital, New York, USA) [23]. MCF-7 cells were transfected (50% confluence) for 24 h with 1 µg of plasmid using FuGENE 6 reagent transfection (Promega, Madison, WI, USA). The selection of successful transfected cells was obtained by supplementing the culture media with 400 µg/ml of zeocin (InvivoGen, San Diego, CA, USA) for 3 weeks and changing medium every 3–4 days. Then cell clones were obtained and characterized. Only the clone number 3 (MCF-7 CAT 3) was selected for this study.

### Induction of cell resistance to oxidative stress

A Resox cell line was established from wild-type human breast cancer MCF-7 cell line, which was purchased from ATCC, as previously reported [18]. Briefly, oxidative stress-resistant Resox cells were made by exposing MCF-7 cells chronically to increasing concentrations of ascorbate/menadione (Asc/Men) during 6 months. To avoid the development of islets of resistance, which could arise from cooperation between cells, the cells were trypsinized approximately every 2 weeks and seeded into new flasks. After selection, the cell line was stabilized in drug-free medium for 1 month.

### siRNA transfection

Dharmafect reagent 1 was used for transfecting siRNA against catalase (ON-TARGET plus SMART pool siRNA) in all cell lines, according to the protocols provided by Dharmacon (Lafayette, CO, USA). Transfections were performed on cells at 50% confluence for 24 h, with a 0.1 μM siRNA solution. All experiments were performed 48 h after transfection. siRNA-mediated catalase knockdown experiments were compared to those displayed by transfecting a siRNA control (scrambled sequence) under the same experimental conditions described above.

### MTT assay

The effects of Asc/Men, arsenic trioxide or hydrogen peroxide on cell metabolic status were assessed by following the reduction of MTT (3-(4,5-dimethylthiazolyl-2)-2,5-diphenyltetrazolium bromide) to blue formazan [24]. Blue formazan crystals were solubilized with DMSO and the coloured solution was subsequently read at 550 nm. Results are expressed as % of MTT reduction compared to untreated control conditions.

### Immunoblotting

At the indicated times, cells were washed twice with ice-cold PBS and then resuspended in RIPA lysis buffer supplemented with 1% Protease Inhibitor Cocktail (Sigma-Aldrich) and 3% Phosphatase Inhibitor Cocktail (Calbiochem, Merck KGaA, Darmstadt, Germany). Electrophoresis of proteins (20 µg), electroblotting and further procedures were done as reported elsewhere [17]. Protein bands were detected by chemiluminescence using the ECL detection kit (Pierce, ThermoScientific, Rockford, IL, USA). When appropriate, bands obtained from Western blot analysis were quantified using ImageJ software (http://rsb.info.nih.gov/ij/).

### Transcriptional activity of human *catalase* promoter

Plasmid pGL3-Basic containing the human *catalase* gene promoter − 1518/+ 16 was kindly provided by Professor M. Nenoi (National Institute of Radiological Sciences, Chiba, Japan) and all constructions strategies were previously described [25]. The transcription start (+ 1) is located 73 bp upstream of the initiation codon [26]. The purified fragment containing human catalase promoter was excised from pGL3 pCAT plasmid by *Kpn*I and *Bgl*II restriction enzymes (Fermentas, Rockford, IL, USA), and ligated into pGL4.15 plasmid (Promega, Madison, WI, USA). For stable transfection, DNA transfection was carried out by using the Xtremegene HP reagent (Roche Applied Science Diagnostics, Mannheim, Germany). Stable transfectants and clones (selected by limiting dilution in a 96-well plate) were selected by supplementing the culture media with 100 µg/ml of Hygromycin B (Invivogen, SanDiego, CA, USA) [17]. Luciferase activities were measured in accordance with manufacturer’s instructions (Luciferase Assay System, Promega, Madison, WI, USA) using a Victor™X2 luminometer (Perkin Elmer, Waltham, MA, USA). Normalized luciferase activity corresponds to measured light units that were normalized to protein concentrations using BCA protein kit (ThermoScientific, Rockford, IL, USA).

### Quantitative reverse transcription-polymerase chain reaction

Total RNA was isolated using Trizol (Invitrogen) according to the manufacturer’s instructions. RNA was reverse-transcribed using Primer Script RT reagent Kit with gDNA Eraser (Takara BIO INC, Kusatsu, Shiga, Japan). Real-time PCR was performed using the SYBR Premix Ex Taq RNAse H + kit (Takara), and analyzed using the Bio-Rad detection system (Bio-Rad, Hercules, CA, USA). The samples were first incubated 5 min at 95 °C, followed by 40 cycles of 10 s at 95 °C and 30 s at 60 °C. The results were calculated (formula: 2-(Ct catalase-Ct EF1)) and matched to the control samples. The primers were produced by Sangon Biotech (Shanghai, China): catalase (sense primer 5′-ccagaagaaagcggtcaagaa-3′; antisense primer 5′-gagatccggactgcacaaag-3′), EF1 (sense primer 5′-cttcactgctcaggtgat-3′; antisense primer 5′-gccgtgtggcaatccaat-3′).

### Flow cytometry

For ROS detection, cells were incubated at indicated time with 10 μM CM-H2DCFDA (Molecular Probes, Rockford, IL, USA) for 20 min. The cells were harvested, washed twice with PBS, and analyzed by flow cytometer (Gallios; Beckman Coulter, Brea, CA, USA).

### Data analyses

All experiments were performed at least 3 times and groups were compared by ANOVA test using GraphPad Prism software (San Diego, CA, USA). Two-way ANOVA test was used to analyze the dose–response curves.

## Results

### Synergistic cytotoxic effect of arsenic trioxide and ascorbate/menadione in breast cancer cells

**Fig. 1:**
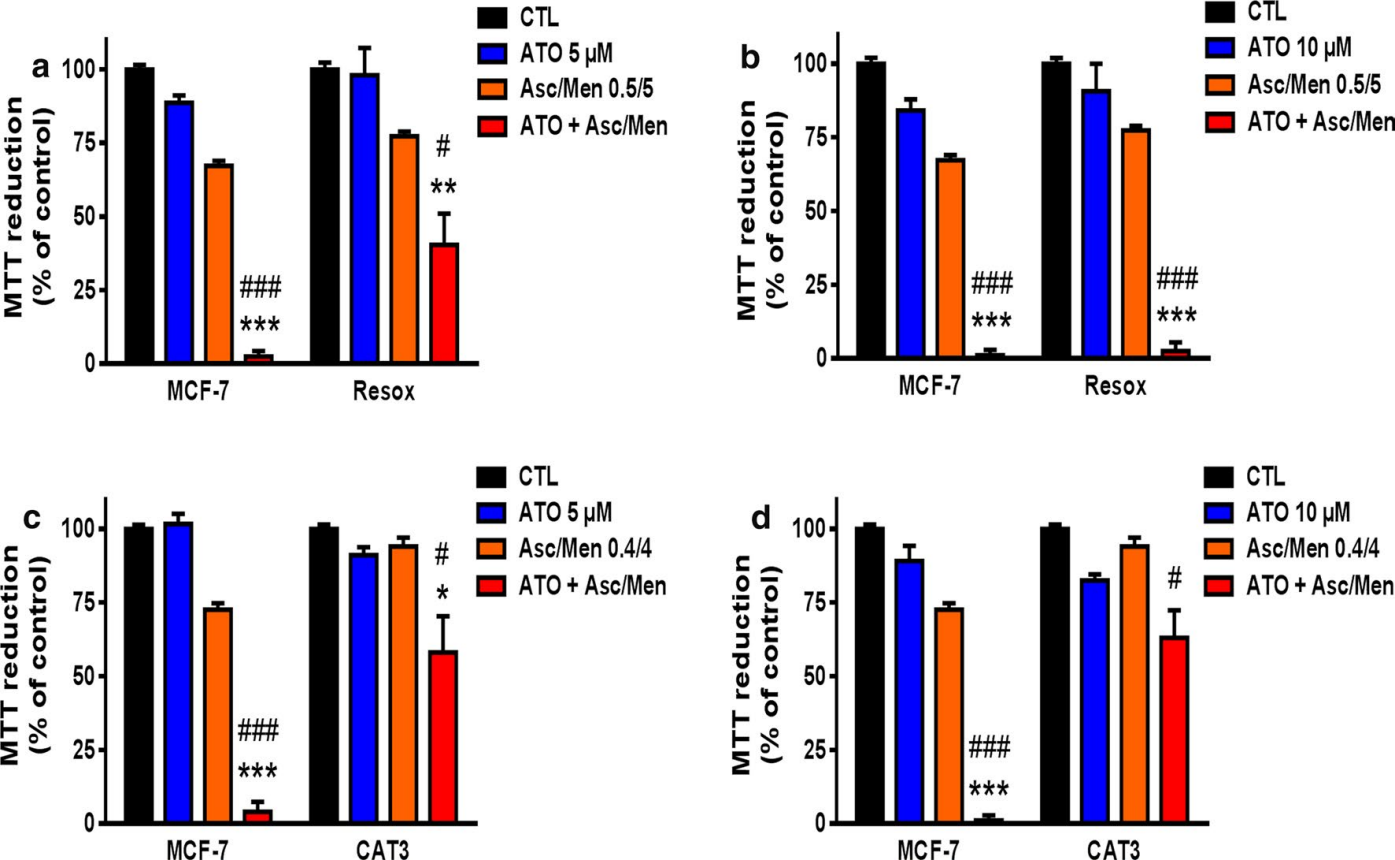
Arsenic trioxide, in combination with ascorbate/menadione, enhances breast cancer cell cytotoxicity. **a, b** MCF-7 (from ATCC) and Resox were incubated 24 h with 5 or 10 μM arsenic trioxide (ATO) with and without Ascorbate 0.5 mM/Menadione 5 μM (Asc/Men). Cytotoxicity was evaluated by using MTT assay (n = 3). **c, d** MCF-7 (from ECACC) and CAT3 cells were incubated 24 h with 5 or 10 μM arsenic trioxide (ATO) with and without Asc/Men (0.4 mM/4 μM), cytotoxicity was evaluated by using MTT assay (n = 3). Data are mean ± s.e.m. Groups were compared using one-way ANOVA followed by Tukey post hoc test for (**a**–**d**). *p value < 0.05; **p value < 0.01; ***p value < 0.001 versus ATO. ^#^p value < 0.05; ^###^p value < 0.001 versus Asc/Men

Preliminary experiments performed in our laboratory using mixtures of Asc/Men with various chemotherapeutic drugs (cisplatin, 5-fluorouracil, doxorubicin, paclitaxel and etoposide) showed that the combination of ATO + Asc/Men was the most effective treatment to overcome this resistance (data not shown). Therefore, we investigated the effect of ATO + Asc/Men on the following mammary cancer cells: MCF-7, CAT3 and Resox cells. Figure 1a–d shows that ATO (5 and 10 µM) enhanced the cytotoxic effects of sub-lethal doses Asc/Men in MCF-7. Such synergistic effect was also observed but to a lesser extent in catalase-overexpressing MCF-7 cells (CAT3 and Resox cells). In addition, the effect of Asc/Men in combination with 10 μM ATO was more pronounced in Resox cells compared to CAT3 cells (Fig. 1b, d).

**Fig. 2:**
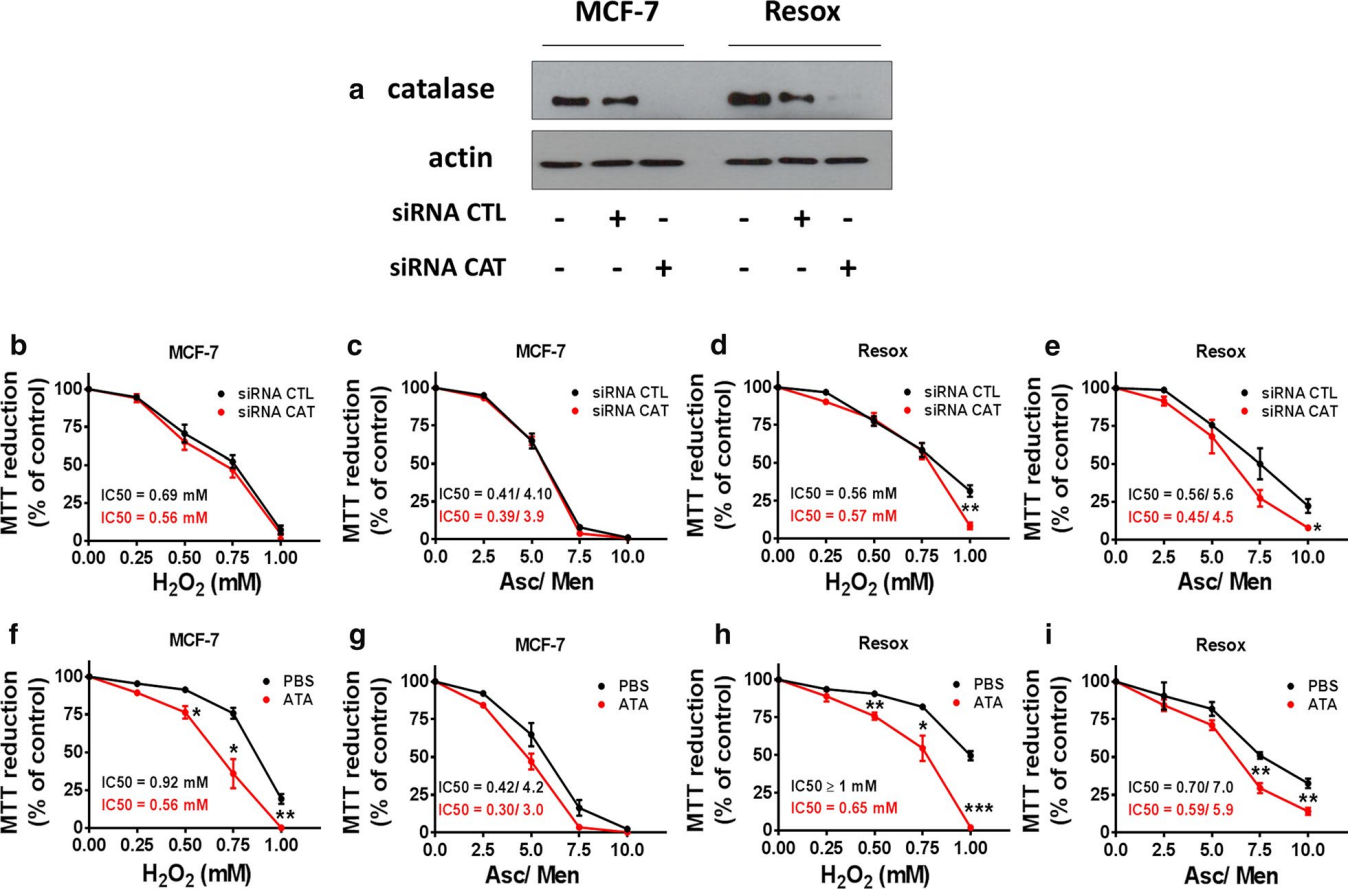
*Catalase* gene silencing and catalase activity inhibition sensitized Resox cells to ascorbate/menadione treatment. **a** Efficiency of *catalase* gene silencing (siRNA) in breast cancer cell lines was evaluated by western blot. **b**, **c** MCF-7 cells were transfected 48 h with control (siRNA CTL) or catalase siRNA (siRNA CAT). Then, cells were incubated with various concentrations of H_2_O_2_ (mM) or with indicated menadione at μM concentrations associated with ascorbate in a ratio Asc/Men 1:100. Cytotoxicity was evaluated by MTT. **d**, **e** Resox cells were transfected 48 h with control (siRNA CTL) or catalase (siRNA CAT) siRNA. Then, cells were incubated as reported in B-C. **f**, **g** MCF-7 cells were incubated for 24 h with PBS or 5 mM ATA. Then, cells were incubated as reported in (**b**–**c**). **h**–**i** Resox cells were incubated for 24 h with PBS or 5 mM ATA. Then, cells were incubated as reported in (**b**, **c**). Data are mean ± s.e.m. Groups were compared using two-way ANOVA test for (**b**–**i**). *p value < 0.05; **p value < 0.01

The key role of catalase in the acquisition of cell resistance against a pro-oxidant treatment was studied by applying a double experimental approach: genetic invalidation via siRNA against catalase and pharmacological inhibition using aminotroazole (ATA), a well-known inhibitor of catalase activity [27]. Genetic invalidation of catalase in MCF-7 and Resox cells reaches 85–95% efficacy of protein expression decrease (Fig. 2a). In MCF-7 cells no changes in sensitivity to increasing concentrations of H_2_O_2_ or Asc/Men in a ratio of 100/1 were observed (Fig. 2b, c). On the other hand, an increased sensitivity to higher doses of H_2_O_2_ and Asc/Men was observed upon catalase knockdown by siRNA in Resox cells (Fig. 2d, e). Regarding the effect by ATA, a much more increase of sensitivity was observed in both cells against H_2_O_2_ exposure (Fig. 2f–h) while a less marked effect was seen in cells incubated with Asc/Men (Fig. 2g–i). Resox cells appear then as more sensitive most likely due to a higher catalase expression at basal levels as compared to parental MCF-7 cells.

Given the pro-oxidant effect exerted by ATO (Fig. 1), the potentiation of its cytotoxicity when associated with Asc/Men, reinforces the validity of a pro-oxidant approach to remove cancer cells. The question about the underlying ATO mechanism thus explaining the enhanced cytotoxicity was therefore raised.

### ATO decreases catalase expression in Resox cells

Since catalase is a key antioxidant enzyme and ATO enhances the sensitivity of catalase-overexpressing cells to Asc/Men (Fig. 1a–d), we further investigated the regulation of the expression of catalase by ATO.

**Fig. 3:**
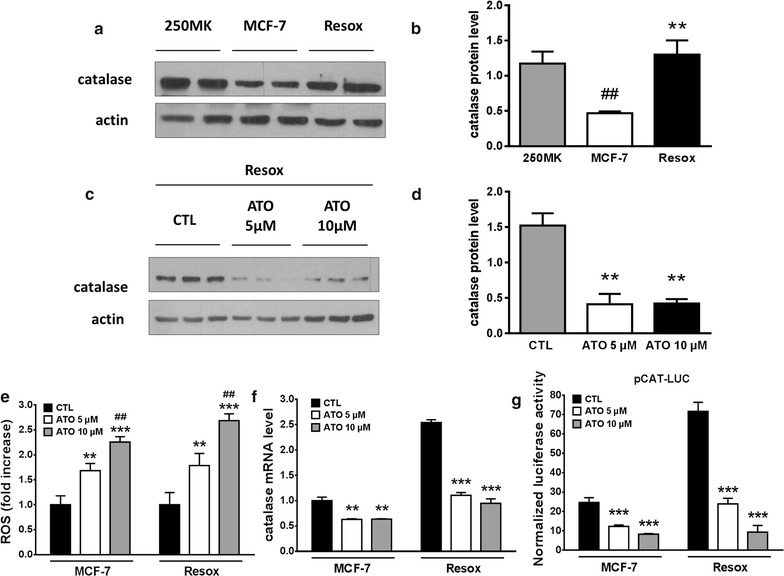
Arsenic trioxide decreases catalase protein level in Resox cells by diminishing *catalase* gene transcription. **a**, **b** Catalase protein levels were compared and quantified in human mammary 250 MK cells, MCF-7 and Resox cells. ^##^p value < 0.01 versus 250MK cells. **p value < 0.01 versus MCF-7 cells. **c**, **d** Catalase protein levels were evaluated and quantified by immunoblotting in Resox cells after ATO treatment (24 h). **p value < 0.01 versus cells not incubated with ATO (CTL). **e** ROS levels were quantified by flow cytometry with CM-H2DCFDA probe. ROS levels were compared to their respective control (CTL). **p value < 0.01; ***p value < 0.001 versus cells not incubated with ATO (CTL). ^##^p value < 0.01 versus cells incubated with 5 μM ATO. **f** Catalase mRNA were measured by qRT-PCR and compared to untreated MCF-7 cells. **p value < 0.01; ***p value < 0.001 versus cells not incubated with ATO (CTL). **g** Normalized luciferase activity was measured in MCF-7 and Resox cells stably transfected with the plasmid pCAT − 1518/+ 16 after ATO incubation for 24 h (n = 3). ***p value < 0.001 versus cells not incubated with ATO (CTL). Data are mean ± s.e.m. Groups were compared using one-way ANOVA followed by Tukey post hoc test for (**b**, **d**–**g**)

We observed that catalase is down-regulated in MCF-7 cells when compared to normal mammary epithelial 250MK cells but its expression is highly enhanced in oxidative stress-resistant Resox cells (Fig. 3a, b). Interestingly, ATO significantly decreases the amount of catalase in Resox cells (Fig. 3c, d) and increases ROS levels (Fig. 3e), likely explaining the enhanced cytotoxicity previously observed (Fig. 1a, b).

Because previous results suggested that alteration of *catalase* gene transcription is the main regulator of catalase expression in cancer cells, we first explored the hypothesis that ATO decreased the transcriptional activity of *catalase* gene promoter.

### ATO decrease catalase expression by controlling the transcriptional activity of its promoter in breast cancer cells

Figure 3f, g shows that ATO represses, in a dose-dependent manner, mRNA transcription and transcriptional activation of human *catalase* gene in both MCF-7 and Resox mammary cancer cell lines. Such repression of transcriptional activity may also explain the differences observed in CAT3 and Resox cells (shown in Fig. 1). Indeed, the difference between them is that Resox cells, after chronic exposure to Asc/Men, acquired a higher transcriptional activity of the endogenous *catalase* promoter, whereas catalase expression in CAT3 cells is the result of a plasmid transfection controlled by SV40 promoter. Thus, in addition to catalase activity, this may explain why CAT3 cells are less sensitive to ATO when its concentration was increased from 5 to 10 µM (Fig. 1c, d). However, CAT3 cells showed an increased sensitivity to the association ATO + Asc/Men compared to the drugs alone. We thus cannot exclude other targets of ATO in these breast cancer cells to explain our findings.

## Discussion

We have previously reported that the combination of ascorbate/menadione (Asc/Men) has a potent antitumor activity both in vitro and in vivo in different tumor types [21, 28–30]. However, increased protein levels of catalase may induce resistance to this pro-oxidant association in breast cancer cells [14].

Our results show that ATO enhanced the cytotoxicity of Asc/Men in breast cancer cells. As expected, cells overexpressing catalase (Resox and CAT3 cells) were more resistant than MCF-7 cells. Interestingly, the cytotoxic effect by ATO + Asc/Men was higher in Resox cells than CAT3 cells. The reason of such different sensitivity is likely due to catalase activity: indeed, in Resox cells the activity of catalase is about 2.5-fold compared to parental MCF-7 cells [16, 17] while in CAT3 cells it is sevenfold higher than in parental MCF-7 cells [14]. In this context, it has been shown that ATO cytotoxicity was potentiated by high doses of ascorbate in AML and APL cells but it was never demonstrated in solid tumors [31, 32].

Catalase is generally down-regulated in tumor tissues compared with their normal counterparts [15]. Furthermore, catalase expression can be modulated by many different processes including genetic alterations, epigenetics, post-transcriptional and post-translational modifications and transcriptional regulation [15]. In agreement with previous results regarding *catalase* gene transcription as the main regulator of catalase expression in cancer cells, we observed that ATO decreased the transcriptional activity of *catalase* gene promoter. To note that ATO (5 and 20 μM) has been shown to decrease catalase activity and mRNA in human osteosarcoma MG63 cells [33] as well as in AML/APL cells [32, 34]. Moreover, inhibition of catalase sensitized K562 cells to ATO [35] but not AML cell lines [34]. It has been also reported that ATO should not be single administrated to combat solid tumors but it requires some associates to have a better clinical efficiency [36]. Since the activity and expression of catalase are significantly altered in osteosarcoma and breast cancer cells, we enthusiastically encourage the proposal that ATO should be administered in combination with pro-oxidant drugs.

**Fig. 4:**
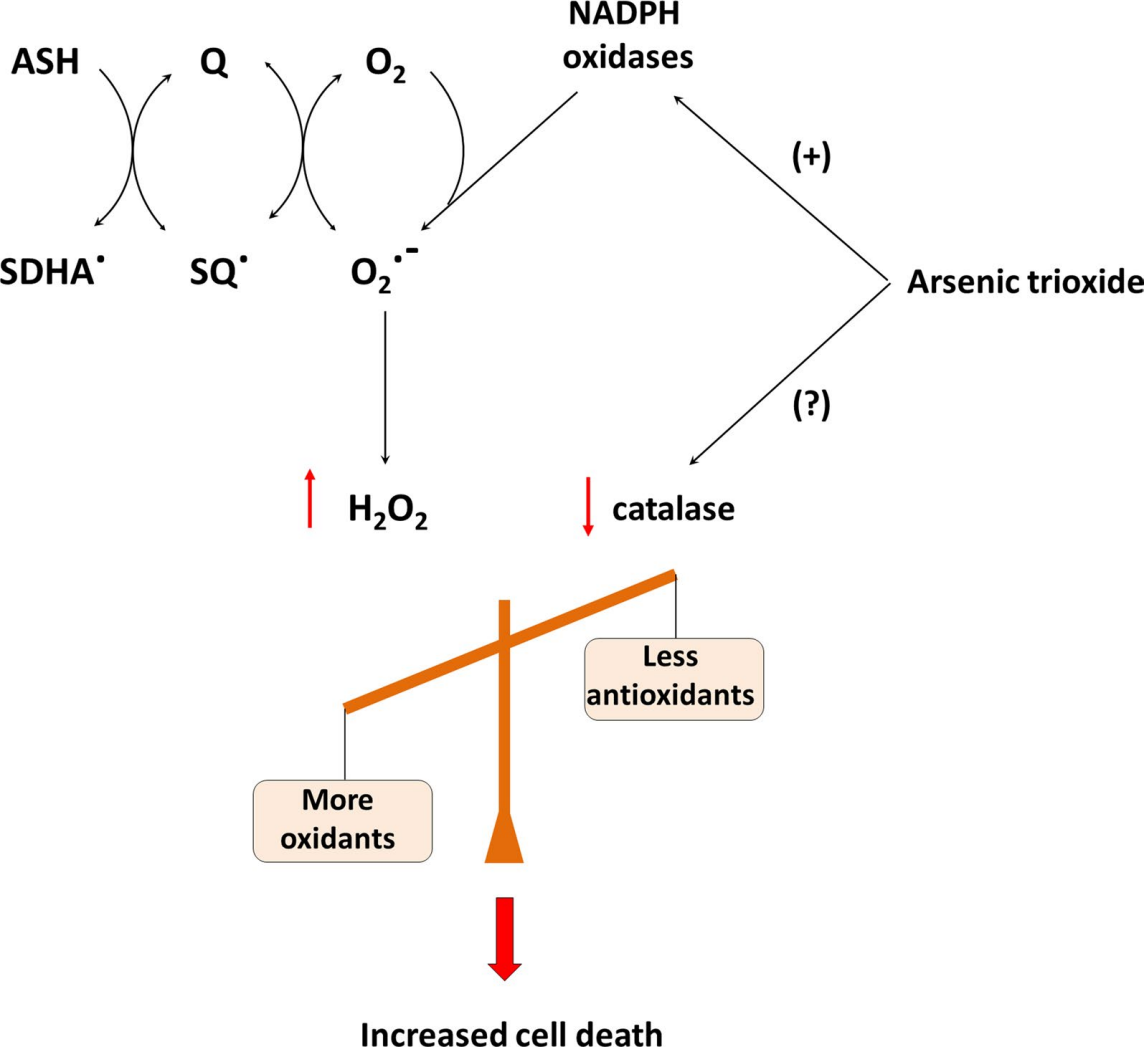
Hypothesis of molecular mechanisms leading to synergistic cytotoxic effects by arsenic trioxide and ascorbate/menadione association. *ASH* ascorbate, *SDHA·* semi-dehydro-ascorbyl radical, *Q*: quinone, *SQ·*: semiquinone radical

Taking together, these results show that ATO sensitizes breast cancer cells by modulating ROS formation and transcriptional activity of *catalase* promoter. Figure 4 shows a scheme with a unified hypothetical view of the synergistic effect mediated by ATO and Asc/Men on a pro-oxidant treatment (Asc/Men). Ascorbate (ASH) enhances quinone (Q) redox cycling, which generates superoxide anion and H_2_O_2_. Meanwhile, ATO also generates ROS (i.e. by activating NADPH oxidases) and was previously considered as ROS-inducing agent in breast cancer cells [37, 38]. Besides this activity, this present study and previous works have shown that ATO decreased catalase expression in solid tumors and leukemia [32–34]. We have shown that JunB induces catalase expression and RARα represses catalase expression [17]. In this regard, it is tempting to hypothesize that ATO may negatively regulate the expression of catalase via RARα [39]. Indeed, in APL leukemia cells, ATO acts mainly on the degradation of the PML-RARα fusion protein, thus explaining the anti-leukemia therapeutic effect by ATO [10, 11]. We will conduct experiments in the future to assess the role of diverse transcription factors in the downregulation of catalase mediated by ATO.

## Conclusion

Arsenic trioxide decreases catalase protein levels by diminishing the transcriptional activity of its promoter. Since catalase is a key enzyme for the resistance against an oxidative stress, ATO could be associated with pro-oxidant drugs to efficiently remove tumor cells.

## Abbreviations

Akt/PKB: protein kinase B
AML: acute myelocytic leukemia
APL: acute promyelocytic leukemia
Asc: ascorbate
ATA: aminotriazole
ATO: arsenic trioxide
ATRA: all-trans retinoic acid
CM-H2DCFDA: 6-chloromethyl-2′,7′-dichlorodihydrofluorescein diacetate
EF1: elongation factor 1
Gpx: glutathione peroxidase
GSH: glutathione
GST: glutathione S-transferase
Men: menadione
PML: promyelocytic leukemia
Prdx: peroxiredoxin
RARα: retinoic acid receptor alpha
ROS: reactive oxygen species
